# Pulmonary toxicity of well-dispersed titanium dioxide nanoparticles following intratracheal instillation

**DOI:** 10.1007/s11051-015-3054-x

**Published:** 2015-06-02

**Authors:** Yukiko Yoshiura, Hiroto Izumi, Takako Oyabu, Masayoshi Hashiba, Tatsunori Kambara, Yohei Mizuguchi, Byeong Woo Lee, Takami Okada, Taisuke Tomonaga, Toshihiko Myojo, Kazuhiro Yamamoto, Shinichi Kitajima, Masanori Horie, Etsushi Kuroda, Yasuo Morimoto

**Affiliations:** Department of Occupational Pneumology, Institute of Industrial Ecological Science, University of Occupational and Environmental Health, 1-1 Iseigaoka, Yahata-nishi-ku, Kitakyushu, Fukuoka 807-8555 Japan; Department of Environmental Health Engineering, Institute of Industrial Ecological Sciences, University of Occupational and Environmental Health, 1-1 Iseigaoka, Yahata-nishi-ku, Kitakyushu, Fukuoka 807-8555 Japan; National Institute of Advanced Industrial Science and Technology (AIST), 1-1-1 Higashi, Tsukuba, Ibaraki 305-8565 Japan; National Sanatorium Hoshizuka Keiaien, 4204 Hoshizuka-cho, Kanoya, Kagoshima 893-8502 Japan; Health Research Institute (HRI), National Institute of Advanced Industrial Science and Technology (AIST), 2217-14 Hayashi-cho, Takamatsu, Kagawa 761-0395 Japan; Laboratory of Vaccine Science, WPI Immunology Frontier Research Center, Osaka University, 6F IFReC Research Building, 3-1 Yamada-oka, Suita, Osaka 565-0871 Japan

**Keywords:** Titanium dioxide, Nanoparticle, Intratracheal instillation, Pulmonary inflammation, Chemokine, Environmental and health effects

## Abstract

In order to investigate the pulmonary toxicity of titanium dioxide (TiO_2_) nanoparticles, we performed an intratracheal instillation study with rats of well-dispersed TiO_2_ nanoparticles and examined the pulmonary inflammation and histopathological changes in the lung. Wistar Hannover rats were intratracheally administered 0.2 mg (0.66 mg/kg) and 1.0 mg (3.3 mg/kg) of well-dispersed TiO_2_ nanoparticles (P90; diameter of agglomerates: 25 nm), then the pulmonary inflammation responses were examined from 3 days to 6 months after the instillation, and the pathological features were examined up to 24 months. Transient inflammation and the upregulation of chemokines in the broncho-alveolar lavage fluid were observed for 1 month. No respiratory tumors or severe fibrosis were observed during the recovery time. These data suggest that transient inflammation induced by TiO_2_ may not lead to chronic, irreversible legions in the lung, and that TiO_2_ nanoparticles may not have a high potential for lung disorder.

## Introduction

Nanoparticles are defined as having a structure such that at least one of their 3 dimensions is about 1–100 nm (ISO). They possess superior characteristics that conventional materials do not have, thus nanomaterials are aggressively researched and developed internationally (ISO 2008). Titanium dioxide (TiO_2_) nanoparticles are one of the representative nanoparticles widely used in cosmetics, sunscreen, and photocatalyst (Kaida et al. 2004). There are some reports of animal studies in which titanium dioxide nanoparticles induced toxic responses, while others have concluded that they did not have toxicity (Skocaj et al. 2011; Iavicoli et al. 2011; Schilling et al. 2010). Consensus on the toxicity of titanium dioxide nanoparticles has not been obtained because of such conflicting reports. Many reports have used acute responses as endpoints for the evaluation of the harmful effects of titanium dioxide nanoparticles in animal studies, but few reports have estimated chronic responses as the endpoint. Considering that the fibrotic and carcinogenic potentials of inhaled materials must be estimated as harmful effects, it is necessary to examine not only the acute responses but also the chronic responses induced by titanium dioxide nanoparticles.

In lung disorders caused by dust, phagocytosis of dust induces infiltration of neutrophils and alveolar macrophages, and persistent or progressed inflammation is likely to cause lung injury and lead to irreversible changes, such as fibrosis and tumor (Nishi et al. 2009; Borm and Driscoll 1996; Kim et al. 2007). Persistent inflammation has been reported in an animal exposure model using asbestos and silica as materials known to have high toxicity (Fubini and Hubbard 2003; Schins 2002). On the other hand, materials with low toxicity, such as micron-sized titanium dioxide, did not cause transient inflammation in some studies but did in others (Morimoto et al. 2013a; Shacter and Weitzman 2002). Therefore persistent inflammation is an important pathophysiology in the formation of irreversible chronic lesion. One of the important cytokines associated with this inflammation is chemokine, in particular, cytokine-induced neutrophil chemoattractants (CINCs), which not only promote neutrophil chemotactic activity but also activate neutrophil function (Shibata et al. 2000). In addition to the persistent inflammation estimated mainly from the acute to sub-chronic phase, it is also important to examine whether or not nanomaterials finally lead to the onset of lung tumor in the chronic phase in order to estimate the toxicity of TiO_2_ nanoparticles.

Therefore, we explored the toxicity of titanium dioxide particles by performing an intratracheal instillation and examining the pulmonary responses not only in the acute phase but also in the chronic phase.

## Materials and methods

### Animals

Male Wistar Hannover rats (11 weeks old) were purchased from Japan SLC, Inc. (Shizuoka, Japan). The animals were bred in the animal research center of the University of Occupational and Environmental Health. Animal handling was carried out according to the guidelines of the University of Occupational and Environment Health.

### Particles

The TiO_2_ (Aeroxide^®^ TiO_2_ P90; nominal diameter: 14 nm) used in this study was purchased from Evonik Degussa Corp. (Nordrhein-Westfalen, Germany). The TiO_2_ nanoparticle suspension was prepared in the manner prescribed by Oyabu et al. (2013). Briefly, TiO_2_ powder was dispersed in sterile purified water by sonication for 45 min at 150 watt using an ultrasonic sonicator (Sonifier 250, Branson, USA) and centrifuged for 20 min at 8900×*g* (Himac CR21, Hitachi, Ltd., Tokyo, Japan). About half of the supernatant was collected. The weight concentration of TiO_2_ in the supernatant was determined and diluted to the instillation concentration with sterile purified water. The size distribution of the TiO_2_ particles was measured by the dynamic light scattering method (DLS-6000AL, Otsuka Electronics, Osaka, Japan).

### Intratracheal instillation study

0.2 mg and 1.0 mg of TiO_2_ nanoparticles were suspended in 0.4 mL of distilled water. Each suspension of TiO_2_ nanoparticles was intratracheally instilled in rats. For a control group, 0.4 mL of distilled water alone was instilled. After instillation, rats were dissected at 3 days, 1 week, 1 month, 3, 6, 12, and 24 months. Ten rats from each group were dissected and divided into two subgroups of five rats each. In the first group, the lungs were inflated with physiological saline with 20 mL at 20 cm H_2_O pressure, and the broncho-alveolar lavage fluid (BALF) was collected from whole lung divided into two to three times. Between 15 and 18 mL of BALF was collected in collection tubes by free fall. In the second group, the lung was divided into right and left lungs. Analysis of cytokine was performed with the homogenized right lung, and histopathological evaluation was performed with the left lung inflated and fixed by 4 % paraformaldehyde.

### Analysis of inflammatory cells in BALF with cytospin

About 10 to 13 mL of BALF was centrifuged at 400×*g* at 4 °C for 15 min. The supernatant was transferred to a new tube and used for measuring the cytokines in the BALF. In order to wash the pellets, they were suspended with PMN Buffer (137.9 mM NaCl, 2.7 mM KCl, 8.2 mM Na_2_HPO_4_, 1.5 mM KH_2_PO_4_, 5.6 mM C_6_H_12_O_6_) and centrifuged at 400×*g* at 4 °C for 15 min. After the supernatant was removed, the pellets were resuspended with 1 mL of PMN Buffer. The cell number in the BALF was counted by Celltac (NIHON KOHDEN CORPORATION, Tokyo, Japan), and cells were splashed on a glass slide using cytospin. After the cells were fixed and stained with Diff-Quik (SYSMEX CORPOTATION, Hyogo, Japan), the number of macrophages and neutrophils was counted by microscopic observation.

### Chemokines and heme oxigenase-1 (HO-1) measurement of lung tissue and BALF

Lung tissue was homogenized with a T-PRE tissue protein extraction reagent (Thermo scientific, MA, USA). Tissue lysates were centrifuged at 21,000×*g* for 10 min, and the supernatant was transferred into a new tube. The protein concentration was measured by Protein Assay (BIO-RAD, CA, USA), using bovine serum albumin to create the standard curve. The total protein concentration was adjusted to a concentration appropriate for each measurement. The concentrations of Rat CINC-1, Rat CINC-2α/β, and Rat CINC-3 in the BALF and lung tissue were measured by ELISA kits, #RCN100, #RCN200, #RCN300 (R&D Systems, Minneapolis, MN), respectively. The concentrations of Rat HO-1 were measured by an ELISA kit, ADI-EKS-810A (Enzo Life Sciences, Farmingdale, NY). All measurements were performed according to the manufacturer’s instructions.

### Hematoxylin and eosin staining of tissue

The lung tissue, which was inflated and fixed with 4 % paraformaldehyde at 20 cm H_2_O pressure, was embedded in paraffin, and 5-μm-thick sections were cut from the lobe, then stained with hematoxylin and eosin.

### Transmission electron microscope (TEM) experimental methods

The lung tissues were fixed by a perfusion system using a 4 % paraformaldehyde solution and were then post-fixed using a 1 % osmium tetroxide solution. They were subsequently dehydrated in ethanol, followed by embedding in epoxy resin. Ultrathin sections were cut with a diamond knife using microtomy. A part of the specimen was stained with a 2 % uranyl acetate solution and a mixed solution of 0.3 % lead nitrate and 0.3 % lead acetate, all at room temperature. Conventional TEM observation was performed with a Hitachi H-7600 (Hitachi, Ltd.) at an accelerating voltage of 80 kV.

### Statistical analysis

Analysis of variance (ANOVA) and Dunnett’s test were applied where appropriate to determine individual differences using a computer statistical package (SPSS, SPSS Inc., Chicago, IL, USA).

## Results

### Characterization of TiO_2_ nanoparticles

The characterization of the bulk particles and dispersed particles was described in a previous paper (Oyabu et al. 2013). Briefly, the specific surface area measured by the BET method (FlowSorb 2300, Shimadzu) and the weighted average surface primary diameter (Sauter diameter) calculated from the specific surface area were 102 m^2^/g and 15 nm, respectively. The aggregation size of the TiO_2_ in the solution as measured by the dynamic light scattering method [(DLS), DLS-600AL, Otsuka Electronics, Osaka, Japan] was 25 nm ± 5 (average ± standard deviation).

### TEM observation of TiO_2_ suspensions

**Fig. 1:**
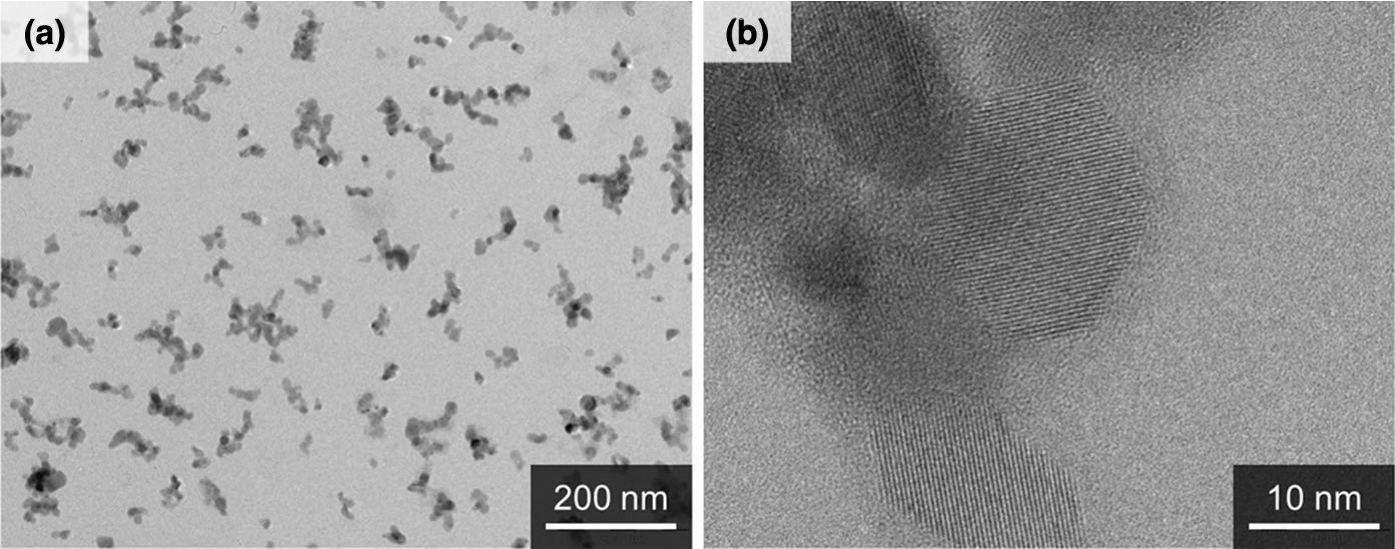
Low magnification (**a**) and high magnification (**b**) TEM images of the TiO_2_ suspensions used in this study

TEM images of the TiO_2_ suspensions are shown in Fig. 1a, b. The TiO_2_ particles were well dispersed and made up of aggregates with sizes between 40 and 80 nm. As can be seen in the high-resolution image, the TiO_2_ particles had a clear crystalline form, and no damage was caused by the preparation processes. The primary TiO_2_ particle size was approximately 20 nm.

### Cell count in BALF

**Fig. 2:**
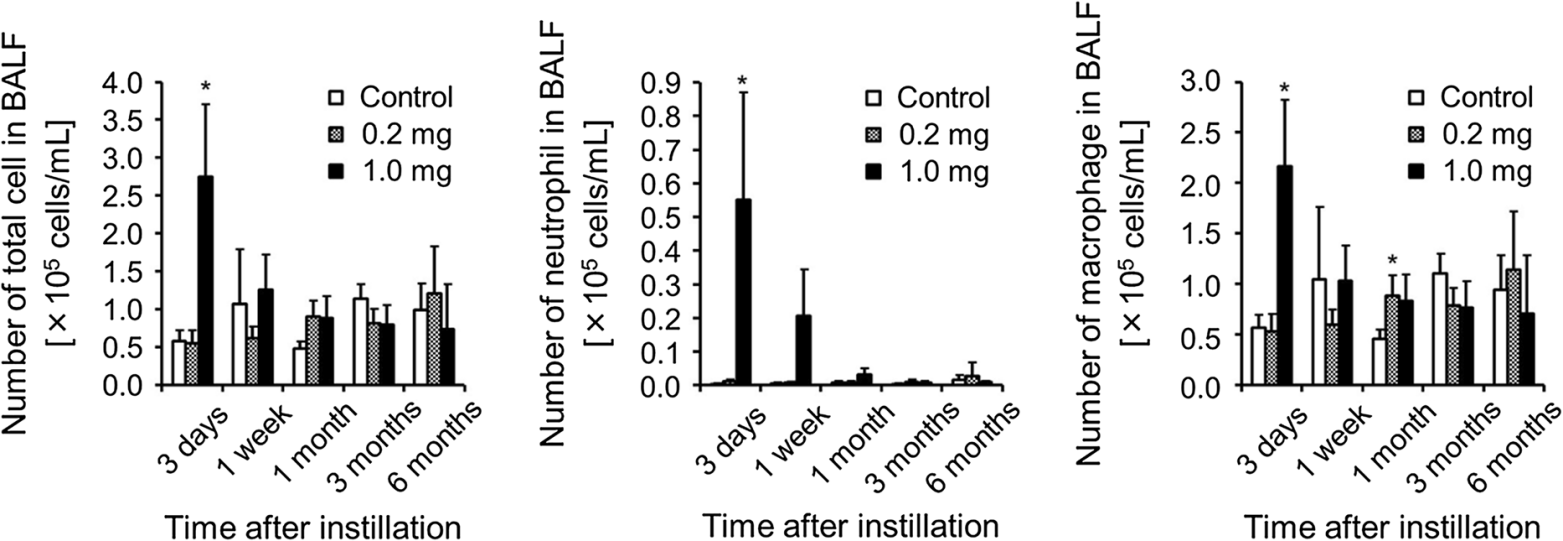
Analysis of BALF after intratracheal instillation of TiO_2_ nanoparticles. Number of total cell, neutrophils, and macrophages in BALF were counted. *Each column* and *bar* represent the mean ± standard deviation of five rats. *Asterisk* indicates significant differences compared with each control (ANOVA, Dunnett T3) (*p < 0.05)

To investigate whether TiO_2_ nanoparticles induce inflammatory cells in BALF, we measured the total number of cells and the macrophages and neutrophils (Fig. 2). In the 1.0 mg-administered group, the number was greatest on day 3. The total number of cells, and macrophages were reduced to the control level after 1 week, while that of neutrophil was reduced to the control level after 1 month. In the 0.2 mg-administered group, TiO_2_ nanoparticles did not cause an increase in inflammatory cells in the BALF.

### Concentration of CINCs in BALF and lung tissue

**Fig. 3:**
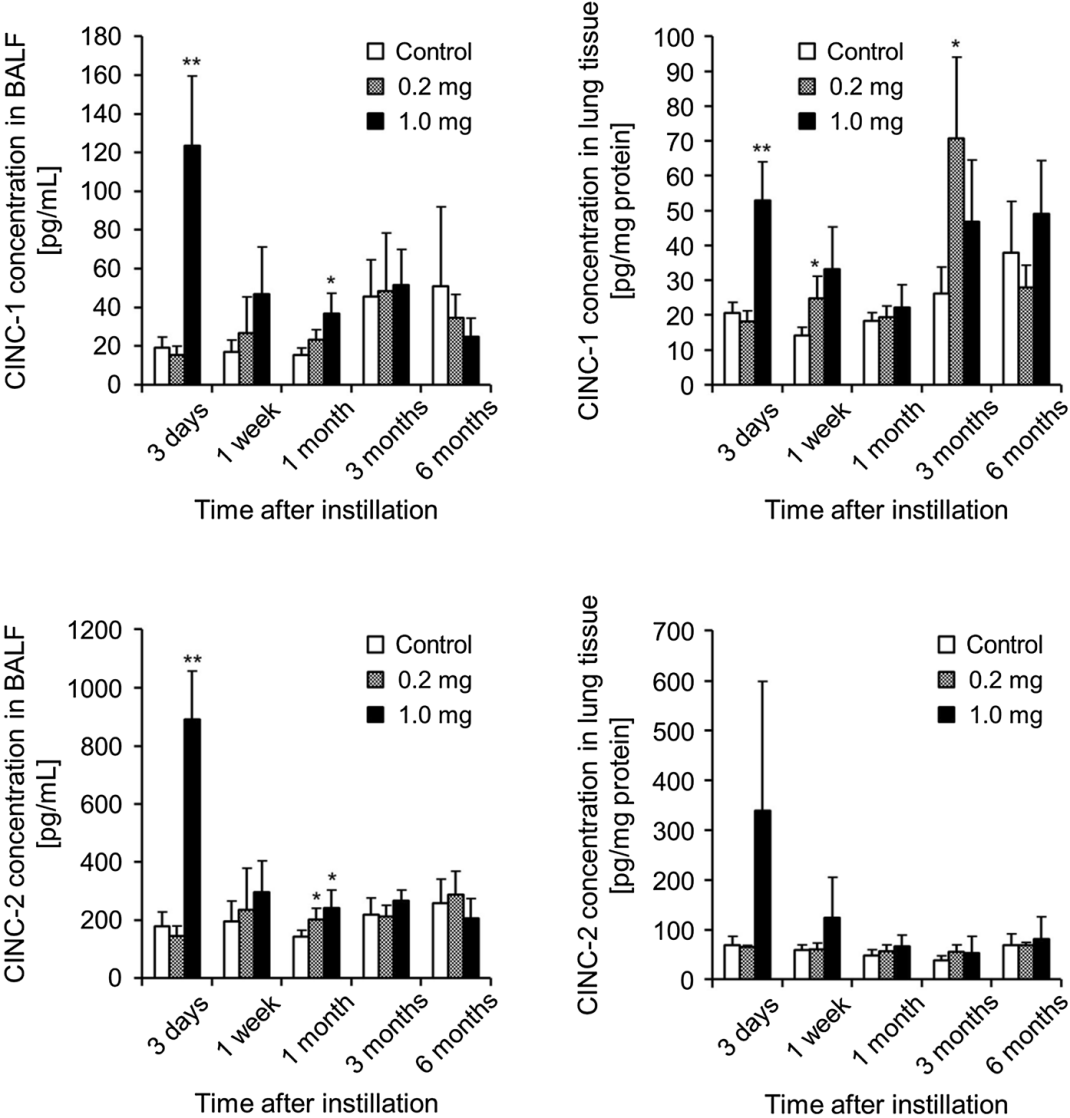
Concentration of CINCs in BALF and lung tissue after intratracheal instillation of TiO_2_ nanoparticles. Concentrations of CINC-1 and CINC-2 in BALF and lung tissue were evaluated with an ELISA kit with specific antibodies. *Each column* and *bar* represent the mean ± standard deviation of five rats. *Asterisk* indicates significant differences compared with each control (ANOVA, Dunnett T3) (**p < 0.01, *p < 0.05)

Because we found a transient increase of inflammatory cells in the BALF, we investigated the chemokines induced by the TiO_2_ nanoparticles. As shown in Fig. 3, the concentrations of CINC-1 and CINC-2 in the BALF increased transiently, reaching a maximum at 3 days in the 1.0 mg-administered group. These inductions decreased by 1 week and were similar to the pattern in the macrophages (Fig. 2). In the 0.2 mg-administered group, by contrast, the TiO_2_ nanoparticles did not affect the concentrations of CINC-1 and CINC-2 in the BALF. In the lung tissue, there were increased concentrations of CINC-1 and CINC-2 in the 1.0 mg-administered group but not in the 0.2 mg-administered group, the same as that in the BALF. The concentrations of CINC-1 in the lung tissue in the 0.2 mg-administered group increased at 3 months, although it is thought that this was not due to the influence of the TiO_2_ nanoparticles. These results were similar to those of our previous reports using TiO_2_ nanoparticles (Oyabu et al. 2013). In that experiment we investigated the concentration of CINC-3 in the BALF and lung tissue as well. We reported a transient increase in the concentration of CINC-3 in the lung tissue caused by TiO_2_ nanoparticles (Oyabu et al. 2013), although the concentrations of CINC-3 not only in the lung tissue but also in the BALF were below the detection limit of the kit.

### Concentration of HO-1 in BALF and lung tissue

**Fig. 4:**
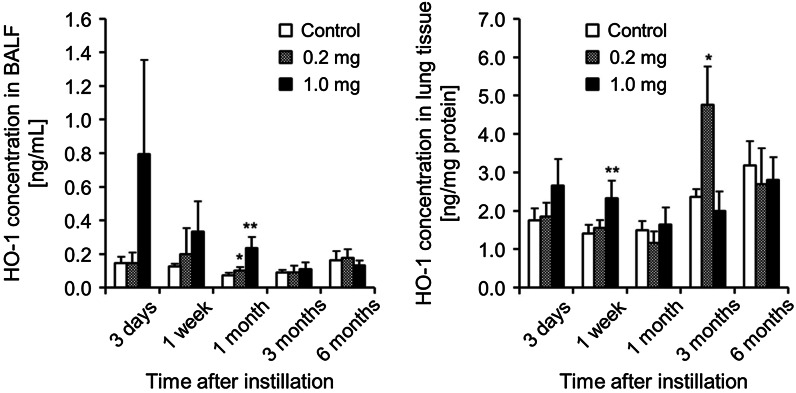
HO-1 concentration in BALF and lung tissue after intratracheal instillation of TiO_2_ nanoparticles. Concentrations of HO-1 in BALF and lung tissue were evaluated with an ELISA kit with specific antibodies. *Each column* and *bar* represent the mean ± standard deviation of five rats. *Asterisk* indicates significant differences compared with each control. (ANOVA, Dunnett T3) (**p < 0.01, *p < 0.05)

It has been reported that nanoparticles often produce reactive oxygen species (ROS), and that cellular HO-1 is induced by ROS to protect the cells from ROS (Wu et al. 2012; Raval and Lee 2010). As shown in Fig. 4, the concentration of HO-1 in the BALF was highest at 3 days in the 1.0 mg-administered group, and a significant increase was observed until 1 month. In the 0.2 mg-administered group, the TiO_2_ nanoparticles did not affect the concentration of HO-1 in the BALF. On the other hand, there were no persist significant differences in the concentrations of HO-1 in the lung tissue in either the 1.0 mg- or the 0.2 mg-administered group, although in the 0.2 mg-administered group in 3 months is higher than in the control.

### Histological findings

**Fig. 5:**
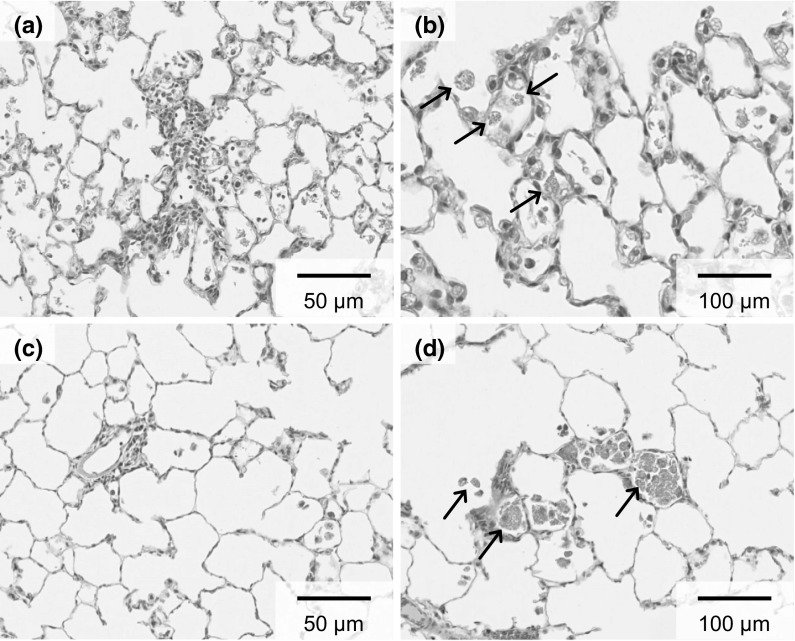
Histological changes in lungs. 3 days post exposure to TiO_2_ in the 1.0 mg-administered group (×200) (**a**) and (×400) (**b**). 6 months post exposure in the 1.0 mg-administered group (×200) (**c**) and (×400) (**d**). *Arrow* particle-laden macrophages containing *brown* particles in their enlarged cytoplasm. (Color figure online)

We investigated the inflammatory response to TiO_2_ by hematoxylin and eosin staining (Fig. 5). No increase of alveolar macrophages was observed in the 0.2 mg-administered group. A mild increase in macrophages was observed in the alveoli in the 1.0 mg-administered group. At 3 days, an accumulation of macrophages and the infiltration of lymphocytes were observed mainly in the alveoli around the alveolar ducts (Fig. 5a). Particle-laden macrophages were scattered in the alveoli (Fig. 5b). The foci of inflammatory cell infiltration and the amount of macrophages decreased with the passage of time after exposure (Fig. 5c). After periods of 1 month or longer, particle-laden macrophages aggregated in the alveoli (Fig. 5d). A histological examination of the lung tissues is summarized in Table 1. No lung tumors or remarkable fibrosis were observed at 24 months post-exposure. In pathological feature in other organs, there are no any tumors in liver, kidney, spleen, testis and brain. Considering these data, we think that small or no amount of TiO_2_ nanoparticles translocate into other organs.

**Table 1:** Histological changes in the lung

Pathological feature	3 days (*n* = 5)			1 week (*n* = 5)			1 month (*n* = 5)			3 months (*n* = 5)		
	Control	TiO_2_ 0.2 mg	TiO_2_ 1.0 mg	Control	TiO_2_ 0.2 mg	TiO_2_ 1.0 mg	Control	TiO_2_ 0.2 mg	TiO_2_ 1.0 mg	Control	TiO_2_ 0.2 mg	TiO_2_ 1.0 mg
Macrophage infiltration	± ~ +	+	+ ~ ++	± ~ +	+	++	±	±	+	±	+	+
Infiltration in interstitial area	− ~ ±	− or +	+	− ~ ±	− ~ +	− ~ +	− ~ +	−	− ~ +	± ~ +	+ ~ ++	±
Fibrosis	−	−	−	−	−	−	−	−	−	−	−	−
Benign tumor	−	−	−	−	−	−	−	−	−	−	−	−
Malignant tumor	−	−	−	−	−	−	−	−	−	−	−	−

Pathological feature	6 months (*n* = 5)			12 months (*n* = 5)			24 months (*n* = 10)		
	Control	TiO_2_ 0.2 mg	TiO_2_ 1.0 mg	Control	TiO_2_ 0.2 mg	TiO_2_ 1.0 mg	Control	TiO_2_ 0.2 mg	TiO_2_ 1.0 mg
Macrophage infiltration	±	±	+	±	±	± ~ +	±	+	± ~ +
Infiltration in interstitial area	−	±	±	−	−	−	−	−	−
Fibrosis	−	−	−	−	−	−	−	−	−
Benign tumor	−	−	−	−	−	−	−	−	−
Malignant tumor	−	−	−	−	−	−	−	−	−

Grade of changes: − none, ± minimum, + mild, ++ moderate, +++ remarked

### TEM observation of lung tissue

**Fig. 6:**
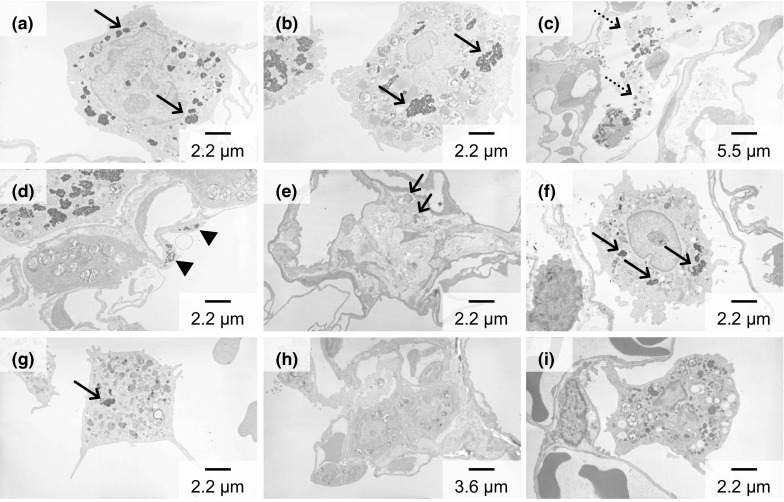
TEM images after exposure to TiO_2_. After 3 days in the 0.2 mg-administered group (**a**) and the 1.0 mg-administered group (**b**–**d**), after 28 days in the 0.2 mg-administered group (**e**) and the 1.0 mg-administered group (**f**), after 6 months in the 0.2 mg-administered group (**g**) and the 1.0 mg-administered group (**h**), after 12 months in the 1.0 mg-administered group (**i**). *Arrow* TiO_2_ aggregates in the alveolar macrophages, *dotted arrow* TiO_2_ aggregates wrapped with alveolar surfactant, arrowhead TiO_2_ in the alveolar cells

TEM images of lung tissue in the 0.2 mg-administered group (Fig. 6a) and in the 1.0 mg-administered group (Fig. 6b–d) 3 days after exposure are shown, respectively. Phagolysosomes with TiO_2_ aggregates were observed in the alveolar macrophages in the 0.2 mg-administered group, as indicated by arrows in Fig. 6a. The same were not observed in the control group. TiO_2_ uptake by alveolar macrophages was also observed in the 1.0 mg-administered group (Fig. 6b). TiO_2_ aggregates wrapped with alveolar surfactant were observed in the alveolar space in the 1.0 mg-administered group (Fig. 6c). It would therefore appear that TiO_2_ was taken up in the alveolar macrophages by endocytosis. Most of the TiO_2_ was observed in the alveolar macrophages, although some was observed in the alveolar cells in the 1.0 mg-administered group, as indicated by arrows in Fig. 6d. There were no TiO_2_ particles in the nuclei of the alveolar macrophages. At 28 days post-exposure, TiO_2_ uptake by alveolar macrophages was observed in the lung interstitium in the 0.2 mg-administered group, as indicated by arrows in Fig. 6e. TiO_2_, indicated by arrows, still remained in alveolar macrophages in the 1.0 mg-administered group (Fig. 6f). Some TiO_2_ was observed in the alveolar macrophages in the 1.0 mg-administered group 6 months after exposure, but most of the alveolar macrophages had not taken up any TiO_2_. Only a small amount of TiO_2_ was observed in the alveolar macrophages (Fig. 6g). A clear TEM image was obtained as a result of the observation of alveolar macrophages in the lung interstitium, with no TiO_2_ being observed (Fig. 6h). Almost all of the alveolar cells and alveolar macrophages were clear at 12 months post-exposure in the 1.0 mg-administered group, and no TiO_2_ was observed (Fig. 6i). Clearance of TiO_2_ is thought to have occurred.

## Discussion

In the present study, 1 mg/rat was used as the maximum dose for the intratracheal instillation. The exposure to 1 mg/rat of fullerene, which is a material with low toxicity and low inflammation, caused transient neutrophil inflammation in rat lung following intratracheal instillation (Morimoto et al. 2010). This neutrophil inflammation estimated by neutrophil counts in the BALF was surely temporary, although the inflammation was slightly sustained. A low dose of fullerene induced no inflammation. We also previously examined the biopersistence of TiO_2_ nanoparticles in rat lung in an intratracheal instillation study, and the clearance of titanium dioxide nanoparticles in the rat lung began to delay at doses exceeding 1 mg/rat (Oyabu et al. 2013). Neutrophil inflammation in the lung was also observed at more than 1 mg/rat. From these data, we speculated that the inflammatory response at doses in excess of 1 mg/rat was caused by additional responses to the excessive dose through delay of the clearance of TiO_2_ nanoparticles, not the original toxicity of the nanoparticles. In the present study, 1.0 mg- and 0.2 mg-administered TiO_2_ nanoparticles caused no or only transient neutrophil inflammation according to analysis of the BALF and the pathological features, suggesting that TiO_2_ nanoparticles have low potential for inflammation. The amount of inhalation exposure, which corresponded to 1 mg/lung as initial lung burden, was speculated using the following formula.

$$\left( {\text{Initial lung burden in rat}} \right) = \left( {\text{exposure concentration of nanoparticle}} \right) \times \left( {\text{tidal volume in rat}} \right) \times \left( {\text{breathing frequency in rat}} \right) \times \left( {\text{exposure hours in day}} \right) \times \left( {\text{particle deposition efficiency}} \right).$$

If we adopted the value as initial lung burden in rat (mg/rat), 1 mg/rat; exposure concentration of nanoparticle (mg/m^3^), 2 mg/m^3^; tidal volume in rat (mL/time), 2.1 mL/time; breathing frequency in rat, 102 times/min; exposure hours in day, 6 h/day; particle deposition efficiency, 0.1 (10 %), the total exposure days at inhalation of TiO_2_ nanoparticles was 64.8 days. We think 1 mg/rat as lung burden may correspond to approximately 13 weeks exposure of with the concentration of 2 mg/m^3^.

We measured the concentration of CINC-1, 2, and 3, representative neutrophil chemotactic factors, in the BALF and in the lung tissue in the present study. It is known that CINC-1, 2, and 3 produced by monocyte, macrophage, and fibroblast have the same function such as activation and chemoattractant of neutrophil (Nakagawa et al. 1994; Mitsuhashi et al. 1999; Hata et al. 2003). It has been reported that different expression patterns of the CINC family have been observed in various inflammation models. There was a high concentration of CINC-1 and CINC-2 in the airway of a rat model of chronic bronchopulmonary infection with *Pseudomonas aeruginosa* (Amano et al. 2000), and Chuang et al. reported that the protein and mRNA expression of CINC-1 was upregulated in an acute lung injury model induced by intratracheal LPS instillation (Chuang et al. 2013). In the 1.0 mg-administered group in the present study, TiO_2_ nanoparticles induced a transient expression of CINC in the rat lung after intratracheal instillation, but not in the 0.2 mg-administered group. In one study, the CINC-1 and CINC-2 expression was upregulated in rat lung with persistent inflammation induced by the intratracheal instillation of nickel oxide nanoparticles (Nishi et al. 2009), while, on the other hand, the results of an intratracheal instillation study using TiO_2_ (micron-size) and fullerene, which is less hazardous to the lung, revealed a mild and transient increase in CINC-1 and CINC-2αβ expression only in the acute phase (Nishi et al. 2009; Morimoto et al. 2010). These findings suggest a positive relationship between pulmonary neutrophil infiltration and CINC expression. Taken together, the similar expression patterns between neutrophil inflammation and chemokines by TiO_2_ nanoparticles in the present study suggest that the expression of CINC may be related to the pulmonary inflammation induced by TiO_2_ nanoparticles.

In the present study, intratracheal instillation of 1.0 and 0.2 mg of TiO_2_ nanoparticles also induced transient and no upregulation, respectively, of HO-1 gene expression, an oxidative stress marker, in the rat lung. Many papers have reported that the upregulation of the gene expression of HO-1 in the lung of dust-exposed animal models accompanied inflammation. Sato et al. reported that cells expressing HO-1 induced by silica were found in granulomatous tissue in mouse lung and found that HO-1 expression in the lungs gradually increased as the disease progressed (Sato et al. 2006). Farina et al. found that intratracheal instillation of PM1 caused an upregulation of HO-1 protein in the lung and acute neutrophil inflammation in mice (Farina et al. 2013). We examined the intratracheal instillation of toner and found that the expression of HO-1 in the lung tissue and BALF was upregulated by the toner in the acute phase and decreased according to a time course, and that this expression pattern of HO-1 was also similar to the pattern of lung inflammation (Morimoto et al. 2013b). The presence of a similarity in the pattern between neutrophil inflammation and HO-1 expression in the present study suggests that the expression of HO-1 in the lung may be related to lung injury induced by TiO_2_ nanoparticles.

Neither 0.2 mg nor 1.0 mg of TiO_2_ nanoparticles intratracheally induced persistent fibrosis or tumor in the rat lung. Heinrich et al. exposed rats to a 10.4 mg/m^3^ average concentration of inhaled TiO_2_ nanoparticles for 24 months and found pulmonary tumor (Heinrich et al. 1995). Pott et al. performed an intratracheal instillation study of TiO_2_ nanoparticles (P25) with an observation time of 24 months, and found that 15, 30, and 60 mg of TiO_2_ nanoparticles caused 52.4, 67.4 and 69.6 % higher incidence of lung tumor, respectively (Pott and Roller 2005). In their studies, the lung burden in rats was thought to be beyond overload. We think that the absence of tumor formation in the present study may mean that the excessive doses caused no additional responses. Significant pulmonary fibrosis in the chronic phase was not found as a pathological feature. In chronic inhalation studies of asbestos and man-made vitreous fibers, the fibers, which caused thoracic tumors in rats, also induced pulmonary fibrosis in rats (IARC). Considering that no fibrosis was found in our present study, we speculate that TiO_2_ nanoparticles may have low carcinogenic potential. Considering that persistent inflammation leads to irreversible lesion, such as tumor and fibrosis, the transient inflammation in the lung accords with the chronic pathological feature in the present study.

## Conclusion

In summary, an intratracheal instillation exposure to TiO_2_ nanoparticle was conducted on rats to examine pulmonary inflammation. Transient pulmonary inflammation was observed with doses of 0.2 and 1.0 mg. No respiratory tumors or severe fibrosis were observed up to 2 years post exposure as pathological findings. These data suggest that TiO_2_ nanoparticles may not have a high potential to cause lung disorder.
